# Do Molecules Tunnel through Nanoporous Graphene?

**DOI:** 10.3390/molecules29143306

**Published:** 2024-07-13

**Authors:** Liudmyla Barabanova, Alper Buldum

**Affiliations:** 1 Department of Chemistry, The University of Akron, Akron, OH 44325, USA; lvb5@uakron.edu; 2 Department of Mechanical Engineering, The University of Akron, Akron, OH 44325, USA

**Keywords:** nanoporous graphene, tunneling, density functional theory

## Abstract

The molecular transport and quantum tunneling of H_2_ and H_2_O molecules through nanoporous graphene is studied using computational modeling and first-principles density functional theory. It is demonstrated that molecules with sufficiently high kinetic energies can tunnel through nanopores. It is also demonstrated that molecules can be trapped in front of a nanopore or behind it. These investigations help us learn the behavior of molecules in and around the nanopores of graphene. They also help us learn the fundamentals of molecular tunneling. We believe nanoporous graphene can play important roles for gas separation and nanofiltration.

## 1. Introduction

Quantum tunneling is a very important phenomenon in quantum mechanics. Its fundamental understanding [1] and its applications [2,3] have always been very attractive to scientists. Tunneling happens when a particle can penetrate through a potential energy barrier which is higher than the particle’s total energy. The tunneling of electrons and protons has always been a focus of interest. Here, we investigate the tunneling of molecules through a potential energy barrier during their transport through an atomically thin membrane. The transport of molecules through a thin membrane has fundamental and technological importance. The possible tunneling of molecules through membranes adds another fascinating dimension to it.

Pristine graphene is impermeable to gases; however, Ga ion bombardment can create holes in the graphene and this can allow gas permeability [4,5,6,7,8]. Nanoporous graphene has great potential to be used in water desalination [9,10,11], nanofiltration [12] and gas separation technology [13,14,15,16,17,18]. Recently, nanoporous graphene was used for in vivo brain signal recording and stimulation [19]. Furthermore, functional nanoporous graphene superlattices and other three-dimensional structures are synthesized for diverse applications [20].

The interaction of water, hydrogen or other fluids with carbon nanostructures has attracted great interest [21,22,23,24,25,26,27,28,29]. The enhanced flow of water through carbon nanotubes was predicted by molecular dynamics simulations [21] and validated by experiments [22,23].

Thiemann et al. showed that fast water molecule transport on carbon is governed by the facile oxygen motion of water molecules [25]. Sabirov performed theoretical studies on synthesized H_2_O@C_60_ endofullerenes [26]. Pizzagalli carried out first-principles molecular dynamics calculations of endofullerenes containing water [27]. Chiricotto et al. studied the wetting behavior of graphitic surface–water interfaces [28]. They found that wettability is due to the fine balance between van der Waals and electrostatic interactions. Y. Xu et al. studied the interaction of charge-neutral and protonated water molecules with graphene oxides, with a focus on epoxide, carbonyl, hydroxyl, and carboxyl groups [29]. They found that these oxygen groups greatly enhance both binding energy and charge transfer between water and graphene.

Here, we investigate the transport and quantum tunneling of H_2_ and H_2_O molecules through graphene nanopores. Atomic models of graphene with different pore sizes are created and molecular transport is studied using first-principles density functional theory. Different orientations of H_2_ and H_2_O molecules and their effects during the transfer are investigated.

It is found that the quantum effect of tunneling can be realized if the total energy of the molecule is sufficiently high and is relatively close to the top of the potential energy barrier. It is also found that there are potential energy wells in front of the nanopore and behind the nanopore in which the molecules can be trapped. The molecular “trap–escape” mechanism is analyzed by calculating the quantized translational energy levels of the H_2_O and H_2_ molecules within the quantum wells.

## 2. Results and Discussion

### 2.1. Nanopore Size and Molecular Orientation Effect on Tunneling Mechanism

**Figure 1:**
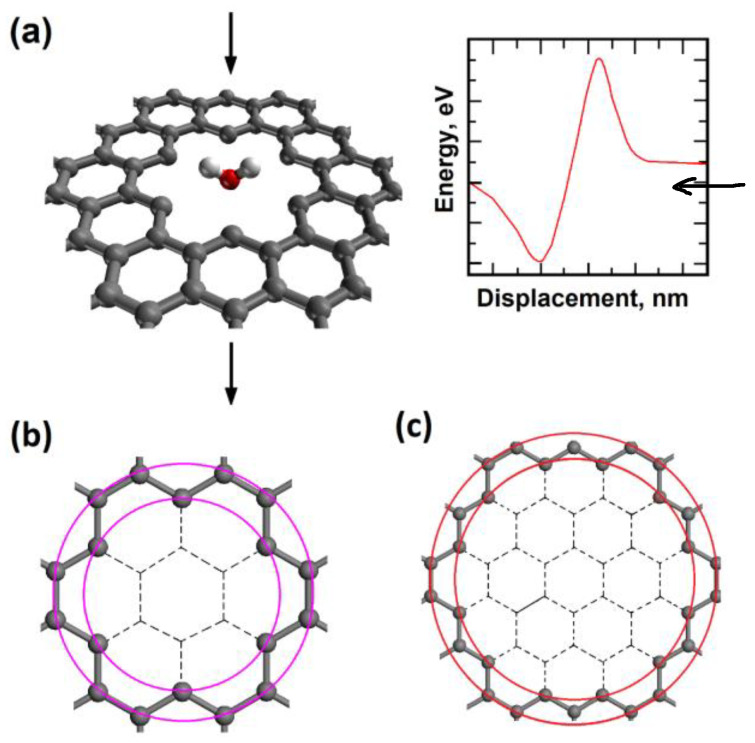
(**a**) Single water molecule with “O atom at the bottom” orientation penetrating the regular-size graphene nanopore; (**b**) regular-size graphene nanopore (inner diameter D_1_ = 0.57 nm; outer diameter D_2_ = 0.75 nm; (**c**) large-size graphene nanopore (inner diameter D_1_ = 1.02 nm; outer diameter D_2_ = 1.24 nm).

**Figure 2:**
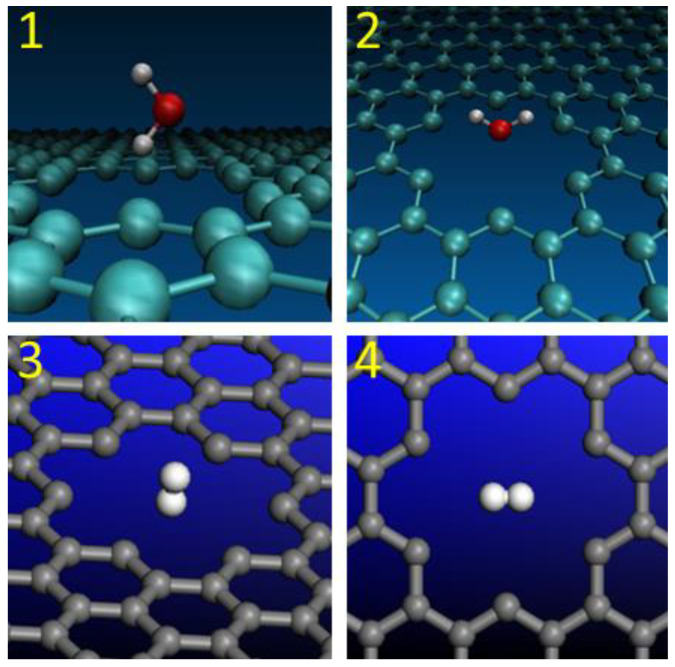
Molecular orientations of H_2_O and H_2_ during the penetration through graphene nanopores: (**1**) H_2_O with “H atom at the bottom”; (**2**) H_2_O with “O atom at the bottom”; (**3**) H_2_ with “vertical” orientation; (**4**) H_2_ with “horizontal” orientation.

**Figure 3:**
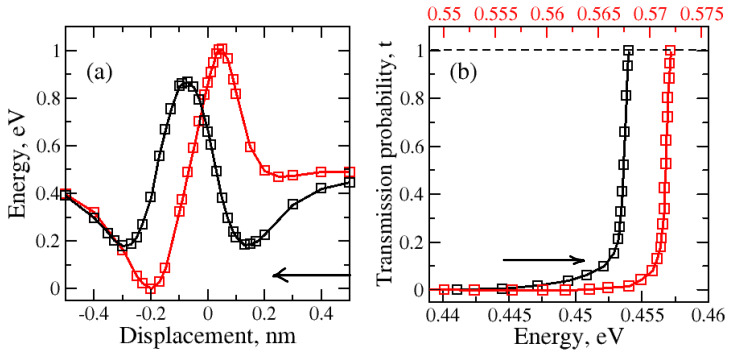
(**a**) Variation in total energy during the water molecule translation through the regular nanopore for two different H_2_O orientations: red—H_2_O (with O atom at the bottom), black—H_2_O (with H atom at the bottom); (**b**) variation in the tunneling or transmission probability due to kinetic energy of the molecule which depends on its temperature. Arrows indicate the direction of molecular motion.

During the one-dimensional displacement of the molecule passing through the nanopore, the total energy changes. This energy decreases first due to adsorption and it increases as the molecule gets closer to the center of the nanopore. As long as the nanopore is not too large, there will be a potential energy barrier for molecular transport. The potential energy barriers for graphene–H_2_O and graphene–H_2_ were calculated. Two different molecular orientations of the water molecule are considered. These are “O atom at the bottom” and “H atom at the bottom” for the regular and large nanopores (see Figure 1 and Figure 2). The tunneling probabilities are calculated using the WKB method which depend on the barrier heights and the kinetic energy of the molecule. The tunneling probabilities for different kinetic energy values are presented in Figure 3.

Initially, the H_2_O molecule was 0.50 nm above the nanopore. The nanopore within graphene was at 0.0 nm. The water molecule passing through the nanopore with an “O atom at the bottom” orientation (Figure 3—red) shows that the total energy first decreases and then increases when the oxygen atom begins to approach the nanopore. The highest increase was observed as the oxygen atom reached the nanopore at 0.0 nm. After another 0.2 nm translation, the total potential energy decreases and this creates a potential energy well (region: 0 nm to −0.5 nm) behind the nanopore. The molecule can be trapped in this potential energy well.

The results for the “H atom at the bottom” orientation passing through the regular-size nanopore are shown in Figure 3. When the first hydrogen atom started to approach the nanopore, the decrease in potential energy caused the creation of the first potential energy well (region: 0.5 nm to 0.1 nm). A significant growth in the barrier height was detected as the oxygen atom came close to the plane of the nanopore. Then, the second hydrogen atom followed the path which led to the second potential well (region: −0.1 nm to −0.5 nm).

The impact of kinetic energy, and thus temperature dependence, on the tunneling probability of the water molecule with “O atom at the bottom” and “H atom at the bottom” orientations passing through the regular graphene nanopore, was computed for the one-dimensional case (Figure 3b—red and black, respectively). The increase in the tunneling probability was observed with the rise in the kinetic energy of the water molecule as the temperature increased. The tunneling occurs at a higher temperature range for the “O atom at the bottom” water molecule orientation than for the “H atom at the bottom” water molecule orientation. The higher temperature indicates that greater kinetic energy is required for the molecule to be able to tunnel through the barrier. This makes the tunneling harder for the “O atom at the bottom” water molecule orientation than for the “H atom at the bottom” water molecule orientation.

The effect of molecular orientation on the possible adsorption mechanism is also clear. The “O atom at the bottom” case has higher adsorption energy if the molecule is trapped on the other side of the nanopore. Also, note the symmetric and asymmetric variation in the total energy due to the molecular orientation.

**Figure 4:**
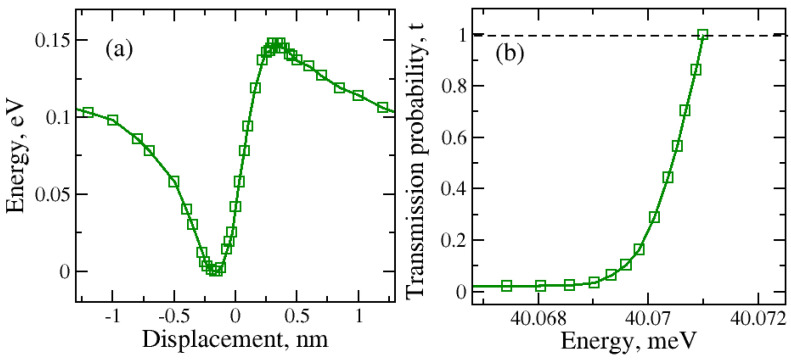
(**a**) Total energy during the water molecule transport through the large graphene nanopore for H_2_O with O atom at the bottom orientation; (**b**) kinetic energy dependence on the tunneling or transmission probability.

The large-graphene-nanopore results are presented in Figure 4. Similar asymmetric total energy variation was observed in the “O atom at the bottom” case.

In contrast with the regular nanopore, a significant difference in the potential barrier height was observed for the larger nanopore. The increase in the graphene nanopore size caused the lowering and also the broadening of the potential energy barrier. For the large nanopore size, tunneling can be observed at lower kinetic energies. In contrast, for both molecular orientations, tunneling through the regular nanopore occurs at higher kinetic energies of the water molecule, enabling it to penetrate through the barrier during the molecular transfer.

**Figure 5:**
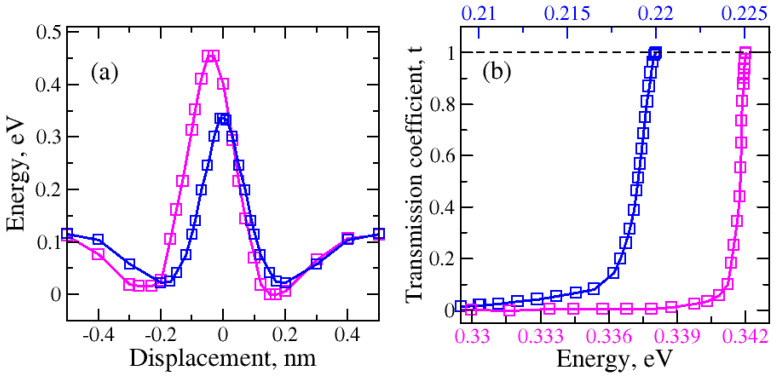
(**a**) Variation in total energy of H_2_ molecule transferred through the regular graphene nanopore for two H_2_ orientations: blue—H_2_ (horizontal), magenta—H_2_ (vertical); (**b**) kinetic energy dependence of the tunneling or transmission probability.

The tunneling of the H_2_ molecule through the regular nanopore was investigated for two different molecular orientations. The horizontal and vertical orientations of H_2_ molecules were considered. The results for these orientations are presented in Figure 5.

Due to its lower potential energy barrier, the H_2_ molecule could tunnel through the nanopore with less kinetic energy and at lower temperatures compared to the H_2_O molecule case. Two potential wells are created in front and behind the nanopore.

By comparing different molecular orientations of H_2_ molecules during tunneling, we found that the H_2_ molecule with “horizontal” orientation created a lower potential energy barrier than the H_2_ molecule with “vertical” orientation. This orientation dependence can be explained by the increased interaction of hydrogen atoms in the molecule with graphene as the molecule approached the nanopore vertically.

We studied the impact of higher kinetic energy and thus the temperature on the tunneling or transmission probability of a H_2_ molecule transferred through the regular nanopore. As it can be seen in Figure 5b, higher kinetic energies and temperatures are required for the tunneling of H_2_ in the “vertical” orientation. Lower kinetic energies and temperatures are sufficient for the tunneling of the H_2_ molecule with the “horizontal” orientation. This makes tunneling easier with “horizontal” rather than with “vertical” orientations.

### 2.2. Total Electron Density of Gas Molecules Inside the Graphene Nanopore

**Figure 6:**
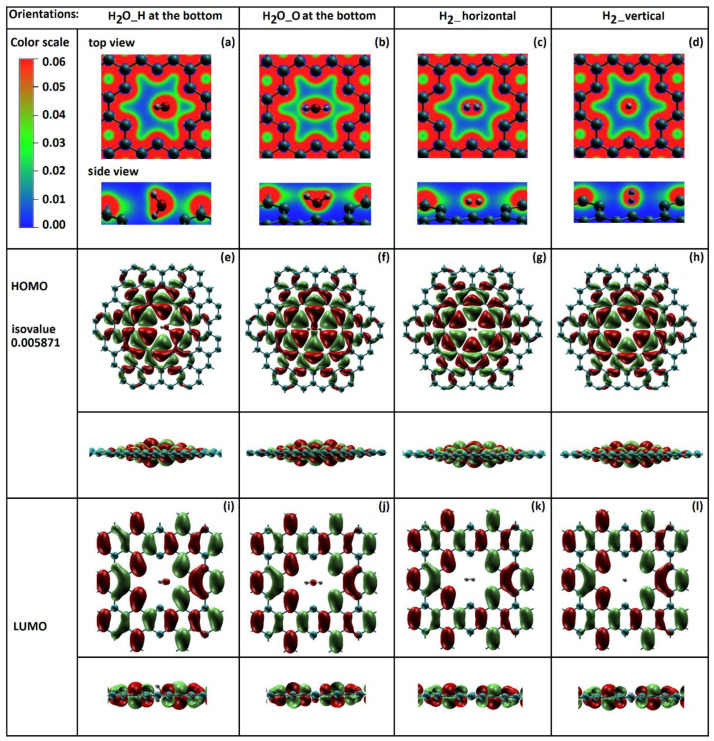
(**a**) Top: the total electron density distribution of (**a**,**b**) H_2_O and (**c**,**d**) H_2_ molecules passing through the regular-size graphene nanopore; bottom: HOMO-LUMO orbital distribution for (**e**,**f**,**i**,**j**) H_2_O and (**g**,**h**,**k**,**l**) H_2_ molecules (isovalue of 0.005871).

To understand the interactions between the molecules and graphene nanopore, we investigate the total electron density of H_2_ and H_2_O molecules inside the regular-size nanopore. Figure 6a–d show the total electron density distribution of H_2_O with “H atom at the bottom”, H_2_O with “O atom at the bottom”, H_2_ with the “horizontal” orientation and H_2_ with the “vertical” orientation, respectively. The top and side views are relative to the graphene plane. We found a significant difference in the total electron density distribution between the H_2_O and H_2_ molecule transfer cases. The higher total electron density between H_2_O molecule and graphene (Figure 6a,b) implies greater molecular orbital overlap and a stronger interaction. There may be also a possible charge transfer between the water molecule and graphene.

For a deeper understanding of the molecular orientation impact on the potential energy barriers, we study the highest occupied (HOMO) and lowest unoccupied (LUMO) molecular orbitals. The molecular orbital isosurfaces for all four orientations are shown in Figure 6 at the fixed isovalue of 0.005871. As can be seen in the HOMO isosurface of the H_2_O molecule with both orientations (Figure 6e,f), an electronic charge transfer between the water molecule and graphene is present. The interaction difference in these two orientations was also observed. In each orientation, oxygen from the water molecule tends to bind to the closest carbon atoms of the graphene nanopore. A weaker interaction was observed for H_2_O_H at the bottom orientation than for the H_2_O_O at the bottom orientation (Figure 6f).

The HOMO isosurfaces of H_2_ molecules (Figure 6g,h) show much weaker interaction with graphene in comparison to the water molecules. This may be due to the smaller molecular size of H_2_. This also validates the results for the lower potential energy barriers for H_2_ molecules.

### 2.3. Molecule Trapping in a Potential Well behind the Graphene Nanopore

When molecules cross the nanopore plane, they can be trapped in the potential well or quantum well behind the nanopore. We investigated the molecular “trap–escape” mechanism from the potential well. Using the harmonic fitting at the bottom of each well, the ground-state zero-point energy was calculated. We utilized the mathematical concept of the Morse potential to calculate the quantized energy levels. The quantization of the translational motion energies was found from the confinement of H_2_O and H_2_ by the graphene nanopore. A well-defined separation of the quantized translational energy levels was observed for both H_2_ and H_2_O due to the small weight of the molecules.

The eigenstates of the translational motion of H_2_O and H_2_ were calculated as the molecules got trapped in the potential well behind the nanopore. For the symmetric total energy potential energy variation cases, the potential well can also be the one in front of the nanopore. The system was treated quantum mechanically for the one-dimensional translational motion with the bound-state approach. The ground-state zero-point energies (E_0_) were calculated at the bottom of each potential well, and the anharmonic part was treated using the Morse potential.

**Figure 7:**
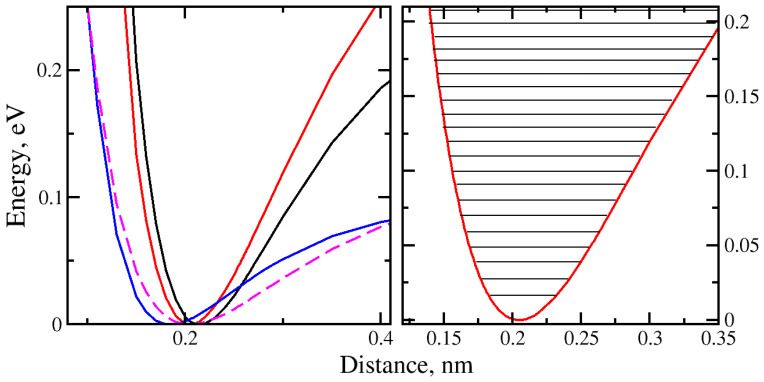
Potential energy wells of water and H_2_ translation through the regular-size membrane: black—H_2_O with “H atom at the bottom”; red—H_2_O with “O atom at the bottom”; magenta—H_2_ with “vertical” orientation; blue—H_2_ with “horizontal” orientation; right panel—quantized energy levels at the bottom of the potential well for H_2_O molecule for “O atom at the bottom” orientation.

The quantized translational energy levels were calculated for the potential energy wells of H_2_O and H_2_ in front of or behind the regular nanopore. The fitted Morse potential energy well obtained by the translational motion of H_2_O with the “H atom at the bottom” orientation, denoted as Model 1, can be observed in Figure 7, represented as a solid black line.

The potential well of the H_2_O molecule with the “O at the bottom” orientation (Model 2) is represented by a solid red line. The potential wells of H_2_ with “vertical” and “horizontal” orientations (Models 3 and 4) are shown in Figure 7 in magenta and blue colors, respectively. Evidence of the molecular orientation effect on the “trapping” mechanism was found.

The deepest potential well was observed for H_2_O with the “O atom at the bottom” molecular orientation (De = 0.3978 eV). The shallowest potential well (De = 0.0962 eV) was for the H_2_ molecule with a “horizontal” orientation in front of or behind the regular nanopore. This implies that, in comparison with H_2_O, significantly less kinetic energy is required for H_2_ to escape the potential well.

The ground-state energies for Models 1 to 4 were calculated with the values of 5.437 meV, 5.677 meV, 9.506 meV, and 11.410 meV, respectively. The smallest ground-state energy was found for H_2_ with a “horizontal” orientation.

The effect of the molecules being trapped behind the nanopore can be discussed by looking at the number of quantized energy levels in the translational degree of freedom that was calculated for each individual potential well corresponding to a particular Model (Table 1). The highest number of the translational energy levels (n) was found for Models 1 and 2 that correspond to the water molecule penetration through the nanopore. This can be explained due to the mass deviation between H_2_ and H_2_O molecules and the increased interaction with the oxygen atom of the H_2_O molecule and the graphene atoms.

Considering the quantum mechanical problem in the one-dimensional case, we calculated the kinetic energy and temperature required for the molecule to escape the potential well. Lower kinetic energies are needed for H_2_ with both orientations to escape the well. Note that k_B_.T at room temperature is roughly 26 meV. Overall, for all the models studied, relatively high temperatures are required for H_2_ and H_2_O molecules to escape the potential wells during transport. This implies that at relatively lower temperatures, the H_2_ and H_2_O molecules are trapped in front of the nanopore or right behind it. On the other hand, there can be molecular collision events and radiation absorption, which can assist their escape.

## 3. Models and Methods

The electronic structures and the potential energy reaction barriers were calculated using ab initio density functional theory (DFT). The quickstep module [30] in the CP2K software package (Version 9.1) was used. Periodic structures can be investigated using this module. The calculations were performed using the Perdew–Burke–Erznerhof (PBE) [31] generalized gradient approximation (GGA) functional for the exchange–correlation term in the Kohn-Sham equations. The DZVP-MOLOPT-SR-GTH [32] basis set and the Goedecker–Teter–Hutter (GTH) [33]-type pseudopotentials were used. The Van der Waals interactions were included using the semi-empirical “DFT + D3” term [34]. The LBFGS [35] optimizer was used for energy minimization to optimize the geometry. The tunneling probability calculations were performed using Wentzel–Kramers–Brillouin (WKB) approximation method [36]. WKB approximation allows one to solve the one-dimensional time-independent Schrödinger equation (*SE*) for the particle encountering the potential barrier, where *SE* takes the form of a simple plane wave when the potential *V*(*x*) is constant:

$$\Psi (x)=Ae^{\pm ikx},\;where\;k=\frac{1}{\hbar }\sqrt{2m(E-V\left(x\right))}\;for\;E\;>\;V(x),$$

$$\Psi \left(x\right)=Ae^{\pm kx},\;where\;k=\frac{1}{\hbar }\sqrt{2m\left(V\left(x\right)-E\right)}\;for\;E\;<\;V(x).$$

For the case when the total energy of the particle is larger than the potential energy of the barrier, *E* > *V*(*x*), the “classical” particle has non-zero energy and is moving freely. The turning point of the classical region is when *E* ≥ *V*(*x*).

In this study, for the constant potential energy of the barrier, the WKB approximation method was applied in the case where the total energy of the particle was less than the potential (*E* ˂ *V*(*x*)). In this case, the “classical” particle is not allowed, but there is a finite probability of the “quantum tunneling” effect as the particle penetrates through the barrier. Buldum and Ciraci used the WKB approximation to investigate the tunneling of xenon atoms from one surface to another in Eigler’s atom switch [37]. Using the WKB approximation method we studied the transmission probability of the H_2_ and H_2_O molecules passing through various graphene nanopore sizes for “classical” and “quantum” regions.

We created an atomistic model of pristine graphene in a rectangular supercell first. The model contains 540 carbon atoms, and the supercell sizes are 3.689 nm × 3.834 nm × 3.00 nm. Periodic boundary conditions are used in all three directions. Next, two different atomic models for two different graphene nanopore sizes were created by using the pristine graphene model. A nanopore is created by removing selected carbon atoms at the center of the rectangular graphene sheet. Nanopores with carbon atoms closest to the pores are shown in Figure 1. The “regular nanopore” model structure has inner and outer diameters of 0.57 nm and 0.75 nm and the “large nanopore” model structure has inner and outer diameters of 1.02 nm and 1.2297 nm, respectively. The carbon atoms at the edge of the nanopores were not hydrogen-ended for the sake of simplicity of the tunneling problem. The chemical nature of nanopores can vary depending on the other atoms attached and can be important for the molecule transport.

H_2_ and water molecules were placed in the middle of each nanopore, and they were translated in the direction perpendicular to the graphene plane. In the models, the graphene plane height was 0.0 nm. Initially, H_2_ and H_2_O molecules were 0.5 nm above the graphene plane, and they were facing the center of the nanopore. The model structure containing a water molecule with its oxygen atom pointing towards the nanopore is shown in Figure 1. The molecule was translated towards the center of the nanopore in discrete steps and the total energy values were calculated using density functional theory. The variation in total energy as a function of molecule height gives us potential energy barriers for molecular transport. Such total energy variation is also presented in Figure 1.

Molecular orientations of H_2_O and H_2_ were also considered during the transfer through graphene nanopores. The first two model structures include the water molecules with “H atom at the bottom” and “O atom at the bottom” molecular orientations. They are presented as Model 1 and Model 2 in Figure 2. The term “at the bottom” implies the specified location of the atom that points towards the nanopore. Model 2 was selected for large-nanopore calculations.

Two different orientations of the H_2_ molecule are presented in Figure 2. They are labelled Models 3 and 4. In the Model 3 structure, the H_2_ molecule with a “vertical” orientation was placed in the middle of the regular graphene nanopore in a way that one of the hydrogen atoms was pointing down towards the center of the nanopore. In the Model 4 structure, the “horizontal” orientation of the H_2_ molecule implies that both hydrogen atoms are placed in the same plane that is parallel to the graphene plane.

## 4. Conclusions

The quantum tunneling of H_2_ and H_2_O molecules through graphene nanopores was studied by performing ab initio calculations based on density functional theory. We found that molecular tunneling depends on the nanopore size, the molecule size and the molecule’s orientation. It also depends on the kinetic energy of the molecule and thus the temperature. For the H_2_O molecule, tunneling occurs at a higher energy and temperature range for the “O atom at the bottom” water molecule orientation than for the “H atom at the bottom” water molecule orientation. Lower kinetic energies and temperatures are sufficient for the tunneling of a H_2_ molecule with a “horizontal” orientation. We also found that there can be potential energy wells in front of or behind the nanopores and H_2_ and H_2_O molecules can be trapped inside these potential wells. Our studies showed that relatively larger kinetic energies and temperatures are required for the molecules to escape the wells. However, molecular collisions and radiation absorption can assist the escape of the molecules. We believe that the unique fundamental differences in quantum tunneling and adsorption behaviors of the molecules would be very helpful for gas separation and nanofiltration technologies.

## Figures and Tables

**Table 1:** The depth of the potential energy wells and the number of quantized translational energy levels of H_2_ and H_2_O molecules.

Models	De, (eV)	n
1	0.2650	50
2	0.3978	70
3	0.1154	12
4	0.0962	8

## Data Availability

Data are contained within the article.
